# Lung Cancer Screening in Head and Neck Cancer: An Opportunity to Increase Screening in Eligible Candidatess

**DOI:** 10.1016/j.atssr.2025.07.006

**Published:** 2025-07-30

**Authors:** Julia Riccardi, Nina D. Ham, Nicole I. Farber, Lisa M. Brown, Marianne Abouyared, Mara B. Antonoff

**Affiliations:** 1 Division of General Thoracic Surgery, Department of Surgery, University of California, Davis, Sacramento, California; 2 School of Medicine, University of California, Davis, Sacramento, California; 3 Department of Otolaryngology, University of California, Davis, Sacramento, California; 4 Department of Thoracic and Cardiovascular Surgery, University of Texas MD Anderson Cancer Center, Houston, Texas

## Abstract

**Background:**

Lung cancer screening is as an effective strategy for early diagnosis in high-risk patients, with proven impact on decreasing mortality. Individuals with malignancies of the head and neck have risk factors similar to those with lung cancer, with the most notable being a shared association with current or previous smoking. We evaluated the prevalence of lung cancer screening candidacy and implementation among head and neck cancer clinic patients.

**Methods:**

Anonymous surveys were collected from patients who presented to the head and neck cancer clinic at a single, tertiary-care center from May 2024 to August 2024. Individuals were queried regarding their candidacy for and experiences with lung cancer screening. Descriptive analyses were performed.

**Results:**

A total of 202 patients were surveyed, with median age of 66 (interquartile range, 15) years. Within this cohort, 77 (38.0%) previously smoked and 15 (7.4%) currently smoked. Among all respondents, 23 (11.4%) met 2021 United States Preventative Service Taskforce criteria for lung cancer screening. Most of the patients (16 [69.6%]) who met criteria had never been offered screening. Seven patients (30.4%) who were eligible for lung cancer screening were previously offered screening and completed it. Of the 25 patients who were screened in the entire cohort, 5 (20%) were diagnosed with lung cancer.

**Conclusions:**

A substantial proportion of head and neck cancer patients are eligible for lung cancer screening. The low levels of screening highlighted in this shared patient population serve as an opportunity for quality improvement, especially given the high frequency of cancer diagnoses among those who received screening.

## Visual Abstract

**Figure:**
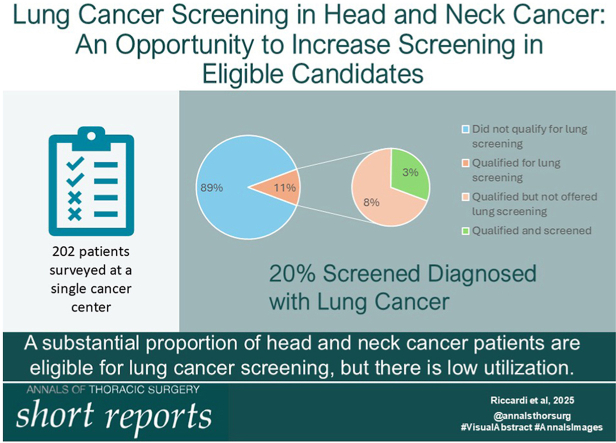

**In Short**

▪ A substantial proportion of head and neck cancer patients are eligible for lung cancer screening. However, few of these patients are offered lung cancer screening.
▪ When these patients are screened, a significant proportion are found to have lung cancer.
▪ Future interventions should be focused on improving lung cancer screening in this population.

Lung cancer remains the leading cause of cancer-related death, with nearly 1.5 million new cases per year.^1^ Although early-stage diagnoses provide a greater chance of cure, most patients present with advanced disease. Much of the delay in diagnoses can be attributed to the lack of cancer-related symptoms with smaller localized tumors, because symptoms do not typically occur until the disease has advanced locally or metastasized to distant sites.^1^

Lung cancer screening is an effective strategy for early diagnosis in high-risk patients, allowing for detection of disease when treatment is likely to be most effective. Moreover, it has been shown to reduce mortality, particularly in high-risk populations.^2^ The substantial life-saving benefits, described by the landmark National Lung Screening Trial, influenced the development of current recommendations for low-dose computed tomographic (LDCT) scans in high-risk individuals.^2^ Up to 80% of lung cancers are attributed to a smoking history.^3^ This knowledge led to the current recommendation, provided by the United States Preventative Services Task Force, for annual LDCT in adults between the ages of 50 and 80 years with ≥20 pack-year smoking history who are currently smoking or have quit in the prior 15 years.

Despite its proven efficacy, lung cancer screening is heavily underused. As few as 16.4% of patients in the United States who meet criteria for lung cancer screening undergo the recommended imaging examination.^4^ Efforts to improve screening rates are strongly needed. Identifying high-risk groups may be an important step toward developing the necessary outreach programs. Patients with cancers of the head and neck have been noted to carry similar risk profiles as those with lung cancer. Analogous to lung cancer, a smoking history is a well-established risk factor for developing a head and neck malignancy.^5^ Prior literature has demonstrated a clear association between head and neck cancer and lung cancer.^6^^,^^7^

Despite the shared risk factors, previous work has demonstrated disappointingly low levels of lung cancer screening among patients with head and neck cancer.^8^ We aimed to evaluate the prevalence of lung cancer screening candidacy and use among patients presenting to the head and neck cancer clinic. The goal of this study was to elicit whether lung cancer screening is underused within this cohort and, if so, aid our institution (and the community at large) in identifying groups and settings that would benefit from targeted education and intervention.

## Patients and Methods

Patients were identified and enrolled in this study at the University of California, Davis Otolaryngology Head and Neck Cancer Clinic. Inclusion criteria required patients to be at least 18 years old and willingness to participate. Exclusion criteria included those who did not submit completed surveys. The study was exempt from review by the University of California, Davis Institutional Review Board.

Anonymous surveys were used to collect patient characteristics, cancer history, and details regarding each patient’s experience with lung cancer screening (Supplemental Appendix 1). The anonymous survey was administered between May 2024 and August 2024. A medical student administered the surveys and was available to answer any questions regarding the survey. Collected variables included age, biological sex, and smoking history, including current vs prior tobacco use, years since quitting, and total pack-years. Patients were also queried on the type of head and neck cancer for which they were being evaluated. Lastly, patients were surveyed regarding their awareness of lung cancer screening candidacy and personal experience with it. The survey was developed and approved by our multidisciplinary team to ensure clarity and readability. The survey was administered to the patient by a member of the research team (N.H.) upon arrival to outpatient appointments at the head and neck clinic. After the surveys had been collected, descriptive analyses were performed to characterize candidacy for screening, its use, and its outcomes.

## Results

A total of 202 patients from the head and neck clinic were surveyed and met inclusion criteria. The response rate was at least 55%. The median respondent age was 66 years (interquartile range, 15 years), and men comprised slightly over half (n = 115 [58.9%]) (Table). Within this cohort, 77 (38.0%) previously smoked, and 15 (7.4%) were currently smoking. Of the 92 who had ever smoked, 73 (79.3%) carried a diagnosis of head and neck cancer. Of the patients with cancers of the head and neck, 16% were currently smoking vs 15% of patients who did not carry diagnoses of head and neck cancers. Those who formerly smoked had quit an average of 20 years (2 weeks-57 years) before completing the survey. Two of the patients in the cohort responded to 1 of the questions on the survey “not sure” and “unknown.”

**Table:** Cohort Demographics, Smoking History, and Lung Cancer Screening Experiences

Characteristic	Surveyed (N = 202)	Active or Former Smokers Who Did Not Qualify (n = 67)	Qualified (n = 23)	Screened (n = 25)
Age, median (IQR), y	66 (15)	70 (17.5)	66 (6.5)	68.4 (10)
Male sex, %	58.9	52	91.3	68
Smoking history, median (IQR), pack-years	0 (15)	13.8 (17.3)	51.5 (21.5)	23 (47.5)
Current smoker, %	7.4	10.4	34.8	20
Average years since quit smoking, median (IQR)	15 (36.3)	26 (27.3)	3 (6.2)	8 (27.8)

IQR, interquartile range.

Among all respondents, 23 (11.4%) met criteria for lung cancer screening according to the 2021 United States Preventative Service Taskforce recommendations.^9^ Seven (30.4%) of these patients were previously offered lung cancer screening and subsequently completed it. Two of these patients (28.5%) were diagnosed with lung cancer. Alarmingly, 16 (69.6%) of the patients who met criteria for lung cancer screening were not offered it (Figure). At the time of the survey, 18 patients (8.9%) did not meet criteria for lung cancer screening but had undergone lung cancer screening in the past. In total, 25 (27.2%) of those who had ever smoked underwent lung cancer screening, and 5 of these 25 patients (20%) were diagnosed with lung cancer.

**Figure 1:**
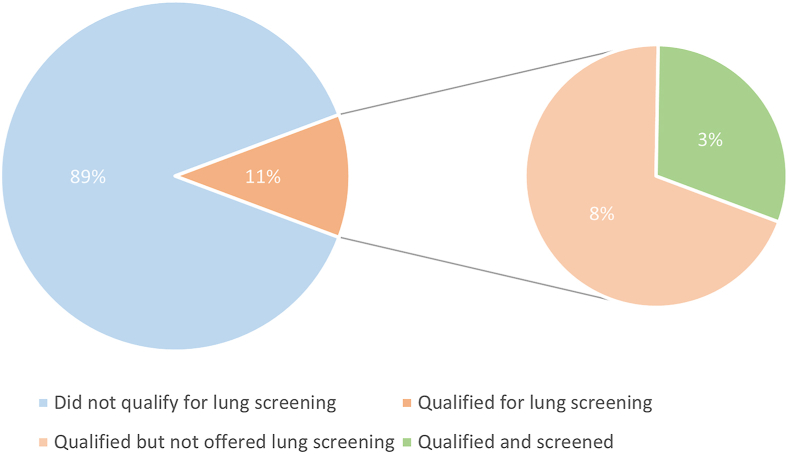
Lung cancer screening in head and neck cancer clinic population.

## Comment

This study demonstrates the significant proportion of head and neck cancer patients eligible for lung cancer screening at a comprehensive cancer center (11.4%). Although the rate of nonsmoker cases of head and neck cancer has increased over time, thought to be secondary to human papillomavirus, smoking remains a major risk factor for head and neck cancer. Despite 11.4% of patients in our cohort meeting criteria for lung cancer screening, only a small proportion are actually being screened for lung cancer, highlighting underuse of screening in this high-risk population. Our results corroborate those of Diaz and colleagues,^8^ who evaluated the use of lung cancer screening in a similar population in the Southern United States. The main difference between these two cohorts is 100% of patients who presented at MD Anderson Head and Neck Cancer clinic had a diagnosis of head and neck cancer vs 79% in this cohort.

Despite its proven mortality benefit within high-risk populations, lung cancer screening remains vastly underused compared with screening methods. Efforts have been made for insurance coverage for these tests with the goal of increasing use. In 2015, the Centers for Medicare and Medicaid Services instituted a national coverage determination within Medicare for lung cancer screening. Under these preventative service benefits, high-risk patients who qualified for LDCT had no out of-pocket expenses.

Primary care providers play a critical role in cancer screening because they are tasked with identifying eligible patients and making the necessary referrals. However, primary care physicians face several key barriers, including insufficient visit time and numerous medical issues that need to be addressed. This issue further supports the need to identify high-yield groups that would benefit from targeted education, with the goals of both educating primary care physicians and decreasing the burden on primary care physicians to individually identify eligible patients. The present analysis suggests that patients with head and neck cancer represent a suitable population for these targeted initiatives.

According to the National Comprehensive Cancer Network guidelines, some but not all head and neck cancer patients are recommended to undergo positron emission tomography or computed tomography chest imaging for initial staging purposes. For example, T1 or 2 glottic cancer does not require chest imaging. The role of chest computed tomography or positron emission tomography scan for surveillance postoperatively in head and neck cancer is controversial, and there is no uniform consensus.

One of the important take-home points from this study is the need to educate head and neck surgeons on lung cancer screening guidelines so they are equipped to appropriately refer patients who qualify for lung screening if they will not require annual cross-sectional imaging that would include the lung fields. We have, importantly, also identified an additional specialty of providers who may aid in the identification of eligible patients outside of the primary care setting. Prior literature has demonstrated lack of provider knowledge to be an important barrier to lung cancer screening.^10^ Future endeavors should focus on whether these barriers can be mitigated by broadening education to subspecialists who see a high proportion of patients eligible for lung cancer screening.

Based on our findings, we propose targeted initiatives to increase education for both providers and patients within the head and neck clinic. The link between head and neck cancer and lung cancer has been well documented. We recognize that patients with head and neck cancer have significantly different expected survival benefits than the patients initially represented by the National Lung Screening Trial. The diminished survival benefit is important to acknowledge, particularly when considering the full range of downstream effects of screening initiatives. However, the United States Preventative Services Task Force recommendations do not exclude patients with other malignancies. Further research is needed to better understand the role that lung cancer screening can have on survivorship within the head and neck cancer population. The present analysis did not intend to elucidate these survival benefits, but rather identify an appropriate and representative target population for engagement.

We recognize the present analysis has several limitations. Our study is survey-based and relies on patient’s ability to recall and provide accurate smoking history, cancer history, and screening history. There are inherent limitations to survey studies. including variable interpretation. To mitigate this potential obstacle, one of the investigatory team was present with each respondent while the survey was completed. Additionally, we recognize that this study was performed at a single, tertiary care, cancer center, optimizing the homogeneity of the cohort, yet with the trade-off of limiting the generalizability of the study outside an academic system. We acknowledge that patients within our health system may have more access to care than most head and neck cancer clinics within the United States.

Our study identified a substantial proportion of patients with head and neck cancer who meet criteria for lung cancer screening. Additionally, it highlights the significant underuse of screening among these patients. The present analysis adds to the growing literature that supports this population to be a suitable target for education and use of lung cancer screening.^8^ Future endeavors should focus on bridging the gap between specialties to aid in optimizing use of lung cancer screening.
