# Supplementary material for: Lung Cancer Screening in Head and Neck Cancer: An Opportunity to Increase Screening in Eligible Candidatess
Source: Ann Thorac Surg Short Rep. 2025 Jul 30;4(1):191–5. doi: 10.1016/j.atssr.2025.07.006 (PMC13100755; doi:10.1016/j.atssr.2025.07.006)
Supplement: Supplementary Appendix [file mmc1.docx]

**Appendix: Lung Cancer Screening Survey**

**Lung Cancer Screening Questionnaire**

*Your participation in this survey is voluntary, and your answers will remain anonymous if you so choose. This questionnaire is not related to your plan of care, so please consult your doctor if you have any health-related questions or concerns.*

Today’s date: _________________________________________________

What is your age? _______________________________________

What is your biologic sex? _______________________________________

Are you a current or previous smoker? _____________________________

If yes, please answer the following questions (*If no, your survey is complete*).

Are you currently a smoker? _____________________________________

If you have quit, how long ago did you last smoke? ___________________

How many total years did you smoke? ______________________________

What is the average number of packs per day that you have smoked? _____

Do you have cancer of the head and neck? If so, what type? _____________

*Lung cancer screening involves a CT scan for people at high risk for lung cancer who do not have any symptoms such as cough or shortness of breath. This screening CT scan is a preventative test to look for cancer.*

Are you aware of who qualifies for lung cancer screening? ______________

Has anyone ever offered you a lung cancer screening CT scan? __________

Have you ever had a CT scan to screen for lung cancer? ________________

Have you ever been diagnosed with lung cancer? _____________________

*Thank you for your participation.*

**Lung Cancer Screening at UC Davis**

The USPSTF recommends annual screening for lung cancer with low-dose computed tomography (LDCT) in adults aged 50 to 80 years who have a 20 pack-year smoking history and currently smoke or have quit within the past 15 years. Screening should be discontinued once a person has not smoked for 15 years or develops a health problem that substantially limits life expectancy or the ability or willingness to have curative lung surgery.

If you are interested in learning more about lung cancer screening or setting up an appointment for your own lung cancer screening, please visit the UC Davis website on lung cancer screening:

*https://health.ucdavis.edu/surgery/specialties/cardio/lung_cancer_screen.html*
